# S100A1’s single cysteine is an indispensable redox switch for the protection against diastolic calcium waves in cardiomyocytes

**DOI:** 10.1152/ajpheart.00634.2023

**Published:** 2024-05-31

**Authors:** Andreas Seitz, Martin Busch, Jasmin Kroemer, Andrea Schneider, Stephanie Simon, Andreas Jungmann, Hugo A. Katus, Patrick Most, Julia Ritterhoff

**Affiliations:** ^1^Molecular and Translational Cardiology, Department of Internal Medicine III, Heidelberg University Hospital, Heidelberg, Germany; ^2^Department of Cardiology and Angiology, Robert-Bosch-Krankenhaus, Stuttgart, Germany; ^3^German Centre for Cardiovascular Research (DZHK), partner site Heidelberg/Mannheim, Heidelberg, Germany; ^4^Department of Internal Medicine III, Heidelberg University Hospital, Heidelberg, Germany; ^5^Informatics for Life consortium, Klaus Tschira Foundation, Heidelberg, Germany; ^6^Center for Translational Medicine, Department of Medicine, Thomas Jefferson University, Philadelphia, Pennsylvania, United States

**Keywords:** calcium, diastolic calcium waves, intrinsically disordered protein, ryanodine receptor 2, S100A1

## Abstract

The EF-hand calcium (Ca^2+^) sensor protein S100A1 combines inotropic with antiarrhythmic potency in cardiomyocytes (CMs). Oxidative posttranslational modification (ox-PTM) of S100A1’s conserved, single-cysteine residue (C85) via reactive nitrogen species (i.e., *S*-nitrosylation or *S*-glutathionylation) has been proposed to modulate conformational flexibility of intrinsically disordered sequence fragments and to increase the molecule’s affinity toward Ca^2+^. Considering the unknown biological functional consequence, we aimed to determine the impact of the C85 moiety of S100A1 as a potential redox switch. We first uncovered that S100A1 is endogenously glutathionylated in the adult heart in vivo. To prevent glutathionylation of S100A1, we generated S100A1 variants that were unresponsive to ox-PTMs. Overexpression of wild-type (WT) and C85-deficient S100A1 protein variants in isolated CM demonstrated equal inotropic potency, as shown by equally augmented Ca^2+^ transient amplitudes under basal conditions and β-adrenergic receptor (βAR) stimulation. However, in contrast, ox-PTM defective S100A1 variants failed to protect against arrhythmogenic diastolic sarcoplasmic reticulum (SR) Ca^2+^ waves and ryanodine receptor 2 (RyR2) hypernitrosylation during βAR stimulation. Despite diastolic performance failure, C85-deficient S100A1 protein variants exerted similar Ca^2+^-dependent interaction with the RyR2 than WT-S100A1. Dissecting S100A1’s molecular structure-function relationship, our data indicate for the first time that the conserved C85 residue potentially acts as a redox switch that is indispensable for S100A1’s antiarrhythmic but not its inotropic potency in CMs. We, therefore, propose a model where C85’s ox-PTM determines S100A1’s ability to beneficially control diastolic but not systolic RyR2 activity.

**NEW & NOTEWORTHY** S100A1 is an emerging candidate for future gene-therapy treatment of human chronic heart failure. We aimed to study the significance of the conserved single-cysteine 85 (C85) residue in cardiomyocytes. We show that S100A1 is endogenously glutathionylated in the heart and demonstrate that this is dispensable to increase systolic Ca^2+^ transients, but indispensable for mediating S100A1’s protection against sarcoplasmic reticulum (SR) Ca^2+^ waves, which was dependent on the ryanodine receptor 2 (RyR2) nitrosylation status.

## INTRODUCTION

S100A1 is an EF-hand calcium (Ca^2+^) sensor protein predominantly expressed in the heart (1). Molecular studies using both gene-deletion and gene-addition strategies characterized S100A1 as a positive regulator of cardiomyocyte (CM) performance (2–7). Lack of S100A1 expression in cardiomyocytes (CMs) disabled the mammalian heart to cope both with acute and chronic hemodynamic stress, and results in accelerated transition to contractile failure (4). By targeting the activity of numerous downstream key effector proteins of the sarcoplasmic reticulum (SR) [ryanodine receptor 2 (RyR2) and SERCA2a], the contractile apparatus (cardiac titin) and mitochondria (complex V), S100A1 plays a critical role as an upstream regulator of the Ca^2+^-controlled contraction-relaxation cycle, energy homeostasis, stress resilience, and rhythm stability of CMs (2, 6, 8, 9). A direct interaction between S100A1 and its various target proteins is a prerequisite for beneficial activity modulation, which is facilitated by the Ca^2+^-activated “on state” of the EF-hand Ca^2+^ sensor (3, 6).

Structural-guided molecular imaging studies raised important questions about the impact of posttranslational modifications (PTMs) on the structure-function relationship of the S100A1 homodimer (10–12). In particular, S100A1’s conserved single-cysteine residue 85 (C85), located in the C-terminal α-helix of each monomer, attracted attention because of its high reactivity toward oxidative PTMs (ox-PTMs). Here, *S*-nitrosylation (SNO) or *S*-glutathionylation (SSG) of C85 emerged as most prevalent PTMs. Subsequently entailing conformational changes, both Ca^2+^ affinity of the two EF-hand domains is enhanced by several orders of magnitude, and the structures of epitopes implicated in target binding are altered (12–15).

No study, however, has yet tested the relevance of C85 ox-PTM for S100A1’s biological function in cardiomyocytes. Given the therapeutic impact of an S100A1 gene-based therapy, we thus aimed to unveil the role of S100A1’s C85 residue as a potential regulatory redox element in CMs. To this end, we tested the hypothesis that C85 allows S100A1 to function as a redox-sensitive Ca^2+^ sensor in CMs, thereby transitioning to a Ca^2+^-activated “on state” already at low diastolic Ca^2+^ concentrations.

Here, we demonstrate that S100A1 is endogenously modified by ox-PTMs and specifically, glutathionylated in the adult heart. We then generated S100A1 protein variants that are unresponsive to ox-PTMs at this site. We demonstrate that C85 is dispensable for enhancing systolic Ca^2+^ fluxes, but indispensable for the protection against diastolic SR Ca^2+^ waves and RyR2 hypernitrosylation in CMs upon catecholamine stress.

Such data advance our understanding of S100A1’s molecular function and, most likely, differentiate for the first time between diastolic redox-sensitive and systolic redox-insensitive molecular mechanisms that are expected to be of equal relevance for S100A1’s therapeutic efficacy in human heart failure.

## MATERIALS AND METHODS

### Experimental Animals

All animal procedures and experiments were performed in accordance with the ethical standards laid down in the 1964 Declaration of Helsinki and its later amendments institutional, guidelines of the University of Heidelberg and received approval from local authorities.

### Materials

Following substances and kits were used: DyLight 680 Maleimide (Thermo Fisher Scientific, 46618), Fura2-AM (life technologies, F-1225), Laminin (Sigma Aldrich, L2020), Medium M199 (Sigma-Aldrich, M7528), Pierce Classic Magnetic IP/Co-IP Kit (Thermo Scientific, 88804), Biotin-maleimide (B1267, Sigma-Aldrich), *N*-ethylmaleimide (E3876, Sigma-Aldrich), ROS-Glo H_2_O_2_ Assay (Promega, G8820). All other chemicals were obtained from Sigma-Aldrich, Germany, if not indicated specifically.

### Generation of Control and S100A1 Variant Adenoviruses

First-generation early gene 1/3-deleted adenoviruses were obtained by the use of the pAdTrack CMV/pAdEasy-1 system as previously described (2, 16). Adenoviruses with C85A and C85S were generated as the wild-type S100A1 adenovirus. Briefly, site-directed mutagenesis of pBS-S100A1 (human cDNA Accession No. X58079, 607 bp) was performed using the following primers: C86A forward, agtggccgctaacaatttcttctgggagaacagttg, and C86A reverse, gaaattgttagcggccactgtgagagcagccac; C86S forward, agtggcctctaacaatttcttctgggagaacagttg, and C86S reverse, aaattgttagaggccactgtgagagcagccac. The S100A1 cDNA was excised using *Hind*III/*Bgl*II and subcloned into the pAdTrack vector. pAdTrack/S100A1 and pAdEasy were used for homologous recombination. The resulting plasmid was linearized using *PmeI* and transfected into Hek293 cells for virus production. The virus was purified using cesium chloride density purification, as previously described (16, 17). Expression of S100A1, C85A or C85S, and green fluorescent protein (GFP) reporter genes was driven by two independent cytomegalovirus (CMV) promoters. The same adenovirus devoid of S100A1 cDNA served as control (AdGFP). Adenoviral titers were determined by anti-hexon immunofluorescent staining in Hek293 and calculated as infectious U/mL.

### Modified Biotin Switch Assay

Biotin switch assay (BSA) was performed with modifications from Aesif et al. (18), Butturini et al. (19), and Li and Kast (20) (mBSA, Supplemental Fig. S1). Hearts from 3- to 4-mo-old male C57BL/6 mice were shock frozen in liquid nitrogen before extraction of proteins in modified HENS buffer (Sigma-Aldrich, 90106, with 1 mM CaCl_2_ and protease inhibitors, mHENS). Protein lysates (2 mg) were used for each reaction. Blocking of free thiols was performed using 40 mM *N*-ethylmaleimide (NEM) in mHENS. After protein precipitation to remove excess NEM, samples were reduced with dithiothreitol (DTT; 40 mM) in mHENS, ascorbate (50 mM) in mHENS with 0.5 mM biotin-maleimide, glutaredoxin (Grx; 13.5 µg/mL, Biomol, Cay31037-100) in 100 mM potassium phosphate buffer at pH 7.5 and 1 mM EDTA, with 1 mM NADPH, 1 mM GSH (Sigma-Aldrich, G4251-5G), 35 µg/mL GSSG reductase (Sigma-Aldrich, G3664-100UN) with 0.5 mM biotin-maleimide.

Protein precipitation and labeling with 0.5 mM biotin-maleimide in mHENS was followed in a separate step. All samples were then precipitated to remove excess maleimide. Samples were resuspended in mHENS and immunoprecipiation (IP) input samples were collected. Of note, we tried increasing ascorbate concentrations (1–50 mM) and various incubation times (1–4 h), as suggested by Forrester et al. (21) but could not detect SNO of S100A1.

For subsequent IP of S100A1, the Pierce Classic Magnetic IP/Co-IP Kit was used. Proteins were eluded by low pH and subjected to nonreducing SDS Page. The control IP to verify specificity of S100A1 IP was performed without mBSA reactions from the same heart samples.

### Neonatal and Adult Rat Cardiomyocyte Isolation

Neonatal rat heart cells were isolated by gradual trypsin digestion from 1 to 3 days old neonatal Wistar rat pups as previously described (4). Pups consisted of both female and male offsprings with natural frequency. Isolated cells were preplated to enrich cardiomyocyte fraction. Cardiomyocytes were plated with a density of 1 Mio cells/mL and cultured for 3 days before viral transduction in medium-199 supplemented with 10% FCS (*days 1* to *2*)/0.5% FCS (from *day 3*) and 10,000 U/mL penicillin-10 mg/mL streptomycin, 1% l-glutamine, and 1 mM CaCl_2_.

Ca^2+^-tolerant adult rat cardiomyocytes were isolated from left ventricle (LV) of male Wistar rats, by a standard enzymatic digestion procedure and cultured as previously described (2). Cardiomyocytes were plated with a density of 50,000 cells/mL on laminin-coated (1:100) fluoro-dishes (World Precision Instruments) or culture plates. Adenoviral transduction of isolated ventricular cardiomyocytes was carried out for 2 h after plating in HEPES-modified medium-199 (M199) supplemented with 5 mM taurine, 5 mM carnitine, 5 mM creatine, 5 mM *N*-mercaptopropionyl glycine, 0.1 µM insulin, 10,000 U/mL penicillin, and 10 mg/mL streptomycin at pH 7.25.

### Intracellular Ca^2+^ Transients

Neonatal or adult rat cardiomyocytes were incubated with of Fura 2-AM (1 µM for 20 min at 37°C followed by 5-min incubation to allow for complete desterification of the dye), and measurements were carried out using an inverse Olympus microscope (IX70) with a UV filter connected to a monochromator (Polychrome II, T.I.L.L. Photonics, Germany) as previously described (2, 22, 23). Cells were electrically stimulated with a biphasic pulse to contract at room temperature and excited at 340/380 nm. Epifluorescence emission was detected at 510 nm, digitized, and analyzed off-line with T.I.L.L.VISION software (v.3.3). Isoproterenol (1 µM) was added after baseline recording when indicated, and measurements were repeated after 5-min incubation.

Data from five consecutive steady-state transients were averaged for analysis of calcium transient amplitude and diastolic calcium levels using a personal automatic detection of transients.

### Sarcoplasmic Reticulum Ca^2+^ Leak Assay: Rapid Pacing

For the rapid pacing, adult rat cardiomyocytes were treated as described earlier. Cardiomyocytes were stimulated for steady-state Ca^2+^ transients at 3 Hz for 20 s alternated by periods of rest of equal duration to allow for the occurrence of spontaneous diastolic Ca^2+^ waves. Isoproterenol stimulation (1 µM) was added to an individual group of CMs and experiments were performed after 5 min of incubation. Ca^2+^ waves were quantified manually by analyzing the number of cardiomyocytes with diastolic Ca^2+^ waves as well as the number of Ca^2+^ waves per cell. Ca^2+^ transient amplitude was analyzed as described earlier.

### Proximity Ligation Assay

Proximity ligation assay (PLA) was performed according to the user manual (olink) and as described previously (23, 24). Neonatal rat cardiomyocytes were plated on laminin-treated (1:200) glass slides as described earlier. Cardiomyocytes were fixed with 4% paraformaldehyde (PFA) solution for 15 min. After washing three times with PBS for 5 min, all cells were permeabilized with 0.5% Triton in PBS for 5 min. After another washing step, unspecific binding was blocked with I-block (Tropix) for 1 h.

Primary antibodies were diluted 1:50 in antibody diluent (S100A1 Acris SP5355P, RyR2 Thermo Fischer Scientific MA3-916, *S*-nitroso-cysteine, Sigma-Aldrich N5411) and incubated overnight. Cells were then washed three times in wash *buffer A* and incubated with the appropriate PLA probes (anti-mouse PLUS and anti-rabbit MINUS, 1:5 in antibody diluent) for 1 h at 37°C. For detection, cells were again washed three times with wash *buffer A*, and ligase mixture (1:50 in ligation buffer) was added and incubated for 30 min. After another washing step, polymerase solution (1:80 in amplification buffer) was added followed by an incubation period of 100 min. Final washing was performed with wash *buffer B*. To distinguish cardiomyocytes, cells were stained with α-actinin (1:300, Thermo Fisher Scientific) and the corresponding fluorescent-labeled secondary antibody (Life Technologies) at room temperature for 1 h each. After a final washing step, cells were mounted with Vectashield (Linaris) and allowed to dry overnight at room temperature before long-time storage at 4°C in the dark. Images were taken with a 10×, 40× objective, or 60× oil immersion objective with an Olympus IX81 microscope. Images were further processed using ImageJ.

### Western Blot Analysis

Protein expression analysis was carried out as described in details elsewhere (7). Briefly, cultured cells were rinsed in PBS and scraped off the dish in lysis buffer (PBS, pH 7.4, SDS 1%, and 1 mM EGTA/EDTA) containing a mixture of 1% (vol/vol) phosphatase inhibitors (Sigma; phosphatase inhibitor mixture I/III) and protease inhibitor (1 tablet/10 mL) (Roche Applied Science; Mini Complete EDTA free protease inhibitor). Protein lysates were subjected to electrophoresis (4–20% tris-glycine gradient gels, ANAMED), transferred to a PVDF membrane (Immobilon FL, Millipore), and probed with appropriate sets of primary antibodies to assess protein levels of S100A1 (Acris SP5355P, 1:1,000 or Abcam ab11428, 1:1,000), GAPDH (Millipore MAB374, 1:30,000), phospholamban (PLB; Thermo Fisher Scientific MA3-922, 1:5,000), Ser16-PLB (upstate 07-052, 1:5,000), Thr17-PLB (Badrilla A010-13, 1:5,000), Ser2808-RyR2 (Badrilla A010-30; 1:1,000), Ser2814-RyR2 (Badrilla A010-31; 1:500), RyR2 (Thermo Fischer Scientific MA3-916; 1:1,000), GFP (Clontech 632375, 1:30,000), and fluorescent-labeled secondary antibodies (LICOR Odyssey, 1:10,000 or Streptavidin 680 DyLight, Thermo Fisher, 21848). Proteins were visualized with a LICOR infrared imager (Odyssey), and quantitative densitometric analysis was performed by applying Odyssey v.1.2 infrared imaging software. Signals were normalized to housekeeping proteins as indicated in the figure legends and did not differ between groups.

### Immunoprecipitation

Immunoprecipitation of RyR2 was carried out as previously described with minor modifications (7, 23). All steps were carried out on ice. Cultured cells were rinsed and scraped off the dish in PBS. After brief centrifugation [13,000 relative centrifugal force (RCF), 4°C for 5 min], pellets were resuspended in nondenaturating lysis buffer, consisting of 1% Nonidet-P40, 10% glycerol, 135 mM NaCl, 1 mM CaCl_2_, and 20 mM Tris·HCl at pH 7.4. Protein lysates were incubated for 30 min at 4°C and centrifuged (13,000 RCF, 4°C for 15 min). Supernatants were diluted to 1 µg/1 µL protein with lysis buffer and rotated with Protein A/G-PLUS-Agarose (20 µL/500 µL; Santa Cruz Biotechnology) for 4 h and centrifuged at 13,000 RCF for 15 min at 4°C to remove protein nonspecifically bound to A/G-Sepharose. The supernatants were then mixed with precipitating antibodies for RyR2 (4 µL) or IgG controls (10 µL, Santa Cruz Biotechnology) and rotated for 8 h at 4°C. Again, A/G-PLUS-Agarose was added and samples were rotated for additional 4 h and centrifuged (13,000 RCF, 4°C for 15 min). Pellets were washed three times with lysis buffer, and 50 µL lysis buffer supplemented with mercaptoethanol (2% vol/vol) was added. Samples were heated at 95°C for 3 min and centrifuged (800 RCF, room temperature for 15 min). The supernatant was transferred in a new vial without aspirating the pelleted beads and instantly resolved by SDS-PAGE.

### Detection of Intracellular Reactive Oxygen Species

Intracellular reactive oxygen species (ROS) were measured using a luminescence-based detection kit as outlined by the manufacturer (ROS-Glo H_2_O_2_ Assay; Promega). Briefly, neonatal rat cardiomyocytes were plated with a density of 400,000 cells/24 well and cultured as described earlier. Isoproterenol stimulation (1 µM) and H_2_O_2_ substrate solution (25 µM) were incubated simultaneously for 20 min. ROS-Glo Detection Solution (50 µL) was mixed with 50 µL cell culture supernatant and allowed to incubate for 20 min before being analyzed in a plate-reader luminometer. For control experiments, cardiomyocytes were treated with 10 µM H_2_O_2_ for 20 min, which resulted in a 50-fold increase in luciferase activity (data not shown).

### Statistics

The numbers of independent experiments are specified in the relevant figure legends. Data are expressed as means ± SE. Statistical analysis was performed with Prism 9.0 or 10.0 software (GraphPad). Statistical comparisons between two groups were performed by unpaired, two-tailed *t* test. Statistical comparisons between three or more groups were performed by one-way ANOVA followed by the appropriate post hoc analysis to determine statistical significance. Details are given in the respective figure legends. The value of *P* < 0.05 was considered statistically significant.

## RESULTS

### S100A1 Is Endogenously *S*-Glutathionylated in the Adult Heart

**Figure 1.**
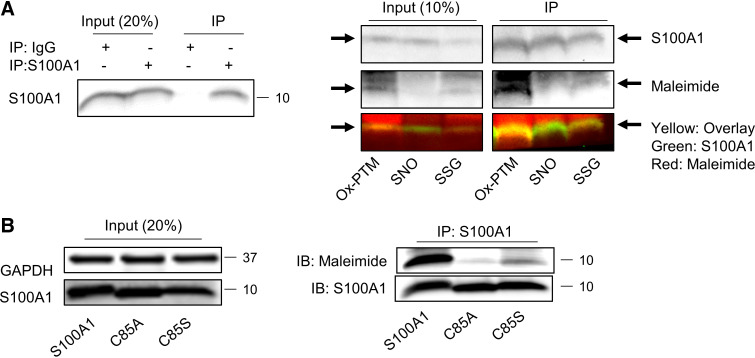
S100A1 is endogenously glutathionylated in vivo. *A*, *left*: immunoprecipitation of S100A1 from mouse heart tissue demonstrating the specificity of S100A1-IP. *A*, *right*: input and S100A1 IP after modified biotin switch assay (mBSA) to detect oxidative posttranslational modification [ox-PTM; reduced with dithiothreitol (DTT)], *S*-nitrosylation (SNO; reduced with ascorbate), and *S*-glutathionylation [SSG; reduced by glutaredoxin (Grx) enzymatic reaction]. Yellow overlay indicates that S100A1 is modified by ox-PTM and SSG. Representative from two experiments. *B*: representative immunoblot of maleimide incorporation after immunoprecipitation of wild-type (WT) and Cys-deficient S100A1 protein from HEK293T cells. Cell lysates were stained with maleimide to label reactive cysteine residues after control, S100A1, C85A, or C85S overexpression. Input (20%) for S100A1 and GAPDH as a loading control (*left*) is depicted ensuring matching S100A1 overexpression. Representative from two experiments.

Biochemical studies suggested that S100A1 can be modified by ox-PTM, including *S*-nitrosylation (SNO) and *S*-glutathionylation (SSG) (10–12). However, these modifications have not been demonstrated in cardiac tissue. To determine if S100A1 is endogenously modified in the heart in vivo, we used a modified biotin switch assay (mBSA) followed by immunoprecipitation of S100A1 (Supplemental Fig. S1) to detect oxidized cysteine modifications, including SNO and SSG (18–20). S100A1 was successfully immunoprecipitated after mBSA, and endogenously modified by ox-PTM (Fig. 1*A*). Surprisingly, we could not detect endogenous SNO of S100A1, as it had previously been proposed in a noncardiac cell type (12), but S100A1-SSG appeared as the prevalent modification (Fig. 1*A*).

To test the functional relevance of this modification, we then generated adenoviruses (Ad) that express human S100A1 protein variants resistant to ox-PTM (C85A and C85S). Functional lack of reactive cysteines was demonstrated by overexpression of WT- and C85-deficient S100A1 variants and labeling with maleimide, which specifically interacts with free thiols. After immunoprecipitation of S100A1, only WT-S100A1 but not C85-deficient S100A1 protein variants were labeled with maleimide (Fig. 1*B*). Of note, a minor fluorescent signal was still visible both in C85A- and C85S-S100A1 overexpressing CMs indicative of endogenous WT-S100A1 protein.

Together, this demonstrates that glutathionylation is the predominant modification of S100A1 in the heart and that C85-deficient S100A1 lacks functional thiols.

### C85A- and C85S-S100A1 Increase Systolic Ca^2+^ Transients in CMs

We next assessed the functional impact of C85-deficient S100A1 protein variants on intracellular Ca^2+^ handling in isolated adult rat cardiomyocytes (ARCMs).

**Figure 2.**
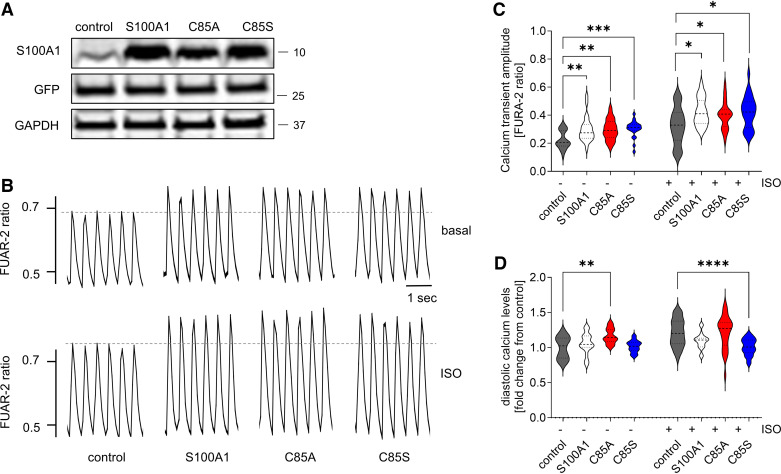
Wild-type (WT) and single cysteine residue 85 (C85)-deficient S100A1 overexpression increase systolic Ca^2+^ transient amplitudes in cardiomyocytes (CMs). *A*: representative immunoblots from control, S100A1, C85A, and C85S-treated adult rat CMs (ARCMs) 24 h after adenoviral transduction demonstrating equal S100A1 overexpression and viral load. Representative from four experiments. *B*: representative tracings of electrically stimulated (2 Hz) steady-state Ca^2+^ transients from control, S100A1, C85A, and C85S overexpressing ARCMs. *C*: quantification of calcium transient amplitude. Overexpression of WT and C85-deficient S100A1 results in increased calcium transients. Note that isoproterenol (Iso) stimulation resulted in a significant increase in calcium transient amplitude in all groups (statistical comparison not shown because of space constraints). *D*: quantification of diastolic calcium levels. Overexpression of C85A results in enhanced diastolic calcium levels, whereas diastolic calcium levels were lower in C85S-treated cardiomyocytes after Iso stimulation. Note that Iso stimulation results in a significant increase in diastolic calcium levels in control cells, but not in any other group (statistical comparison not shown because of space constraints). Data are given as means ± SE; *n* = 29, 22, 21, 21, 29, 19, 27, and 25 of control, S100A1, C85A, C85S, control Iso, S100A1 Iso, C85A Iso, and C85S Iso, respectively. Statistical comparison was performed with one-way ANOVA with Sidak’s multiple comparison post hoc test (*C* and *D*). *P < 0.05, **P < 0.005, ***P < 0.001, ****P < 0.0001.

Adenoviral delivery of WT- and C85A- and C85S-S100A1 resulted in an approximately fivefold increase of each S100A1 protein variant after 24 h (Fig. 2*A*, Supplemental Fig. S2, *A* and *B*). Of note, overexpression levels matched those achieved in previous studies that disclosed the inotropic actions of S100A1 in rodent CMs (23, 25, 26). All overexpressed S100A1 protein variants yielded a significant but indistinguishable increase in Ca^2+^ transient amplitudes under basal conditions (Fig. 2, *B* and *C*). Alike WT-S100A1, the gain of function by C85-deficient S100A1 variants was preserved under βAR stimulation using isoproterenol (Iso) (Fig. 2, *B* and *C*). Diastolic Ca^2+^ levels were enhanced after C85A overexpression under basal conditions. In contrast, diastolic Ca^2+^ levels remained significantly lower in C85S-expressing CMs after βAR stimulation.

In summary, S100A1’s C85 residue seems dispensable for increasing systolic Ca^2+^ transient amplitudes in CMs but has distinct effects on diastolic Ca^2+^ handling.

### C85-Deficient S100A1 Variants Fail to Protect Against βAR-Triggered Diastolic Arrhythmogenic Sarcoplasmic Reticulum Ca^2+^ Waves

Alterations in diastolic Ca^2+^ handling can be caused by several factors, including altered SERCA activity or RyR2 performance (27).

**Figure 3.**
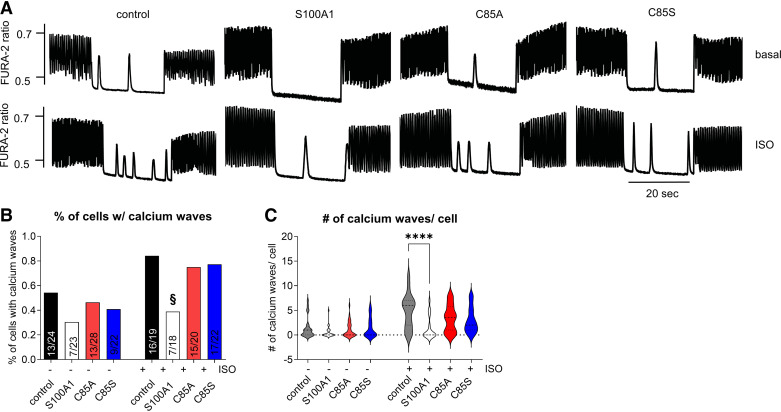
Wild-type (WT) but not single cysteine residue 85 (C85)-deficient S100A1 protects against rapid pacing-induced calcium waves in cardiomyocytes (CMs). *A*: representative tracings of electrically stimulated Ca^2+^ transients from basal and isoproterenol (Iso)-stimulated (1 µM) control, S100A1, C85A, or C85S-treated adult rat CMs (ARCMs). Cells were electrically stimulated (3 Hz) for 20 s followed by a 20-s resting period and restimulation (3 Hz) for 20 s. *B*: percentage of cells with spontaneous calcium waves was quantified. Iso stimulation results in an increase in calcium waves in control, C85A, and C85S-treated cardiomyocytes but not after WT-S100A1 overexpression. *C*: number of calcium waves per cell was quantified. WT-S100A1-treated cardiomyocytes demonstrate fewer calcium waves after Iso stimulation. Iso stimulation results in an increase in calcium waves in control, C85A, and C85S-treated cardiomyocytes but not after WT-S100A1 overexpression (statistical comparison not shown because of space constraints). Data are given as means ± SE; *n* = 24, 23, 28, 22, 19, 18, 20, and 22 of control, S100A1, C85A, C85S, control Iso, S100A1 Iso, C85A Iso, and C85S Iso, respectively). Statistical comparison was performed with χ^2^ test/Fisher’s exact test (*B*) or one-way ANOVA with Sidak’s multiple comparison post hoc test (*C*). §*P* < 0.05, control Iso vs. S100A1 Iso, ****P < 0.0001.

Given the previously reported protective effect of overexpressed WT-S100A1 against diastolic RyR2 dysfunction and arrhythmogenic sarcoplasmic reticulum (SR) Ca^2+^ leakage (23), we subsequently determined the efficacy of C85-deficient S100A1 protein variants on diastolic CM performance. To this end, we used a previously published steady-state rapid pacing (3 Hz) protocol to enhance SR Ca^2+^ load, which enables the formation of diastolic Ca^2+^ waves because of abrupt cessation of the stimulation (Fig. 3*A*).

Under baseline conditions, WT-S100A1 tended to decrease the incidence of the SR Ca^2+^ waves, although this trend did not reach statistical significance (Fig. 3, *A*–*C*). As desired, βAR stimulation by Iso exaggerated the incidence of spontaneous SR Ca^2+^ release that was measured both by the number of CMs with diastolic Ca^2+^ release and number of Ca^2+^ waves per cell (Fig. 3, *A*–*C*). Overexpression of WT-S100A1 completely prevented the βAR-mediated increase in diastolic Ca^2+^ waves in CMs compared with controls, which concurs with previous results (23). Although C85A- and C85S-S100A1 protein variants also increased the systolic Ca^2+^ transient amplitudes during the 3-Hz steady-state pacing period to the same extent than WT-S100A1, both C85-deficient S100A1 protein variants failed to protect CMs against spontaneous and βAR-triggered diastolic Ca^2+^ waves (Fig. 3, *A*–*C*).

Hence, C85 appears indispensable for S100A1’s control of the diastolic RyR2 leakage.

### C85A- and C85S-S100A1 Interact with the RyR2 at Diastolic Ca^2+^ Levels

Failure of C85-deficient S100A1 variants to prevent βAR-triggered RyR2 dysfunction prompted the question of whether C85 substitution might disable S100A1 to interact with the RyR2 and/or unfavorably alter posttranslational modification of the SR Ca^2+^ release channel.

**Figure 4.**
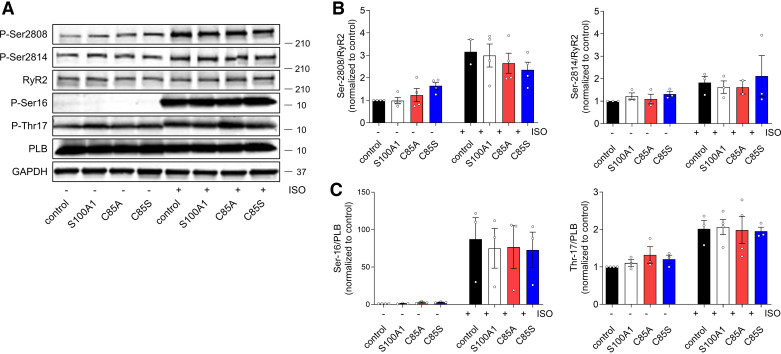
Wild-type (WT) and single cysteine residue 85 (C85)-deficient S100A1 overexpression has no impact on ryanodine receptor 2 (RyR2) and phospholamban (PLB) phosphorylation in cardiomyocytes (CMs). *A*: representative immunoblots for total PLB, PLB Ser-16, and Thr-17, total RyR2, RyR Ser-2808, and Ser-2814 in control, S100A1, C85A-, and C85S-treated adult rat CMs (ARCMs). Quantification shows no differences in RyR2 (*B*) and PLB (*C*) phosphorylation levels between control, S100A1, C85A, and C85S-treated CMs, whereas isoproterenol (Iso) stimulation increases protein kinase A (PKA)-dependent phosphorylation of RyR2 and PLB. Data are given as means ± SE; *n* = 3–4; Statistical comparison was performed with one-way ANOVA with Sidak’s multiple comparison post hoc test.

As previous reports linked protein kinase A (PKA) and CamKII-mediated RyR2 hyperphosphorylation to diastolic gating dysfunction (28, 29), we next determined RyR2 phosphorylation at the corresponding serine-2808 and -2814 sites (Fig. 4*A*). However, none of the overexpressed S100A1 variants mitigated the βAR-induced increase in RyR2 phosphorylation (Fig. 4, *A* and *B*). In addition, PLB was included to capture potential differential activity on βAR downstream signaling in other SR compartments. Again, no differences between the overexpressed S100A1 protein variants on global signaling were observed (Fig. 4, *A* and *C*).

**Figure 5.**
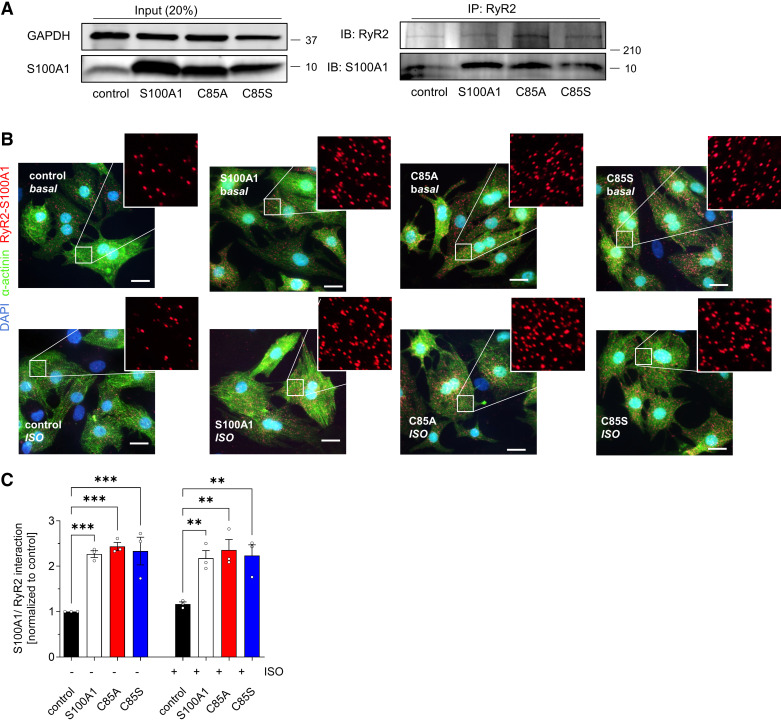
Wild-type (WT) and single cysteine residue 85 (C85)-deficient S100A1 interact with the ryanodine receptor 2 (RyR2). *A*: representative immunoblot for RyR2 and coprecipitating S100A1 protein after RyR2 immunoprecipitation from control, S100A1, C85A-, or C85S-treated neonatal rat cardiomyocytes (NRCMs). *Left*: input (20%) for S100A1 and GAPDH as loading control. *B*: representative proximity ligation assay (PLA) images from control, S100A1, C85A-, and C85S-treated NRCMs showing S100A1/RyR2 interaction (red dots). *Inset*: magnification is twofold. Scale bar represents 20 µm. *C*: statistical analysis shows a twofold increase of the S100A1/RyR2 binding ratio; *n* = 3. Data are given as means ± SE. Statistical comparison was performed with one-way ANOVA with Sidak’s multiple comparison post hoc test (*C*). **P < 0.005, ***P < 0.001.

Furthermore, ox-PTM of C85 might be required for S100A1’s Ca^2+^-dependent interaction with target proteins (6). We thus assessed the capability of overexpressed S100A1 variants to interact with the RyR2 in neonatal rat cardiomyocytes (NRCMs), which exhibited the same inotropic response after overexpression of the S100A1 protein variants (Supplemental Fig. S3, *A–D*). Coimmunoprecipitation analysis demonstrated that C85A- and C85S-S100A1 increased the S100A1/RyR2 ratio as WT-S100A1 under high Ca^2+^ conditions (Fig. 5*A*). We subsequently used the in-cell proximity ligation assay (PLA) to study S100A1/RyR2 binding, as this method enables the detection of protein-protein interactions within their intact subcellular domains at near diastolic Ca^2+^ concentrations in quiescent CMs (Fig. 5*B*). Interestingly, all overexpressed S100A1 protein variants resulted in a significant twofold increase of the S100A1/RyR2 ratio at baseline and after βAR stimulation (Fig. 5, *B* and *C*).

Together, this demonstrates that C85-deficient S100A1 did not change the S100A1/RyR2 stoichiometry in quiescent CMs at systolic and diastolic Ca^2+^ levels, despite persistent altered RyR2 phosphorylation.

### C85-Deficient S100A1 Variants Fail to Abrogate βAR-Induced RyR2 Hypernitrosylation

**Figure 6.**
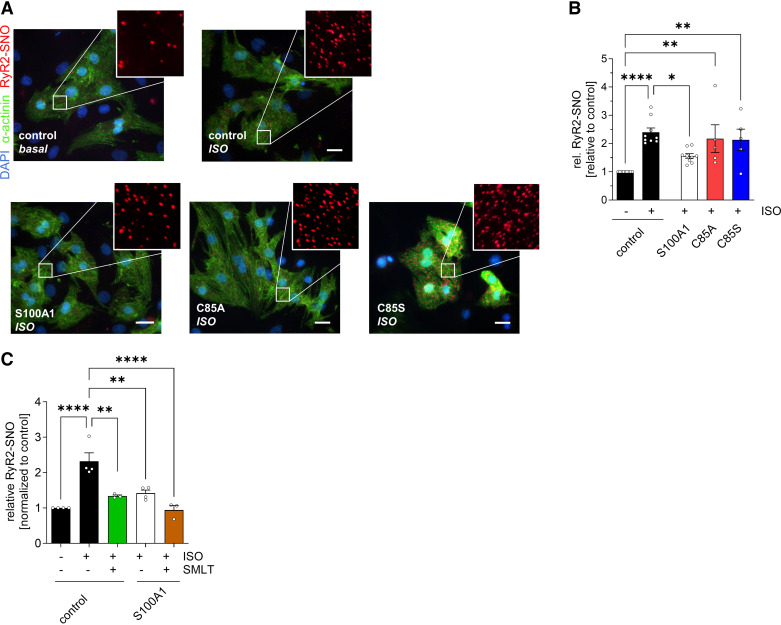
Wild-type (WT) S100A1 prevents ryanodine receptor 2 (RyR2) hypernitrosylation during β-adrenergic receptor stimulation. *A*: representative proximity ligation assay (PLA) images from control, S100A1, C85A-, and C85S-treated neonatal rat cardiomyocytes (NRCMs) showing pan-RyR2 nitrosylation (red dots). *Inset*: magnification is twofold. Scale bar represents 20 µm. *B*: quantification of *A*. RyR2 is hypernitrosylated after isoproterenol (Iso) stimulation, which is prevented after WT, but not after C85-deficient S100A1 protein overexpression; *n* = 5–9. *C*: quantification of RyR2-*S*-nitrosylation (SNO) after *S*-methyl-l-thiocitrulline (SMLT) stimulation in indicated groups. SMLT reduced RyR2-SNO during Iso stimulation in control cardiomyocytes (CMs); *n* = 3–4. Data are given as means ± SE. Statistical comparison was performed with one-way ANOVA with Sidak’s multiple comparison post hoc test (*B* and *C*). *P < 0.05, **P < 0.005, ****P < 0.0001.

Besides phosphorylation, βAR stress has been reported to render the SR Ca^2+^ channels leaky via aberrant hypernitrosylation of distinct reactive cysteines (30, 31). To determine if RyR2-SNO is affected by S100A1, we again used the in-cell PLA assay. βAR stimulation resulted in a significant increase of RyR2-SNO in control cells (Fig. 6, *A* and *B*), as previously demonstrated (30, 32), and WT-S100A1 overexpression suppressed this βAR-mediated RyR2 hypernitrosylation. In contrast, both C85A- and C85S-S100A1 failed to abrogate hypernitrosylation of RyR2 under βAR stimulation (Fig. 6, *A* and *B*). Hence, this is the first evidence that S100A1 can impact the function of a crucial intracellular SR target by modulating the NO-dependent redox-state via its conserved C85 residue.

Nitric oxide synthase (NOS)1 (nNOS)-mediated RyR2-SNO has been shown to modulate cardiac contractility and ventricular arrhythmias (32, 33), so we next investigated a potential role for S100A1 in compartmentalized SR nitric oxide (NO) signaling. Here, inhibition of NOS1 with *S*-methyl-l-thiocitrulline SMLT was sufficient to abrogate Iso-induced RyR2-SNO but did not further reduce RyR2-SNO after WT-S100A1 overexpression (Fig. 6*C*), supporting the role of NOS1-mediated RyR2 hypernitrosylation. S100A1 has been demonstrated to interact with NOS3 (endothelial nitric oxide synthase, eNOS) in endothelial cells thereby modifying vascular NO signaling (24). Since NOS1 rather than NOS3 is targeted to cardiac SR and RyR2 (32), we sought to determine whether WT-S100A1 might interact with NOS1 or modulate its expression. However, we could not provide evidence for WT-S100A1 to interact with NOS1 in CMs assessed by PLA or coimmunoprecipitation (data not shown) and thus could not establish a direct link between S100A1 and NOS1 activation.

Alternatively, to a direct, NOS1-dependent nitrosylation modification, a second pathway that increases oxidative stress in the vicinity of the target has been proposed to regulate nitrosylation of SR proteins (32). To test this alternative hypothesis and assess if S100A1 affects whole cell redox homeostasis, we measured cellular ROS generation in response to Iso stimulation in NRCMs. Neither WT-, C85A-, nor C85S-S100A1 overexpression had an impact on global ROS levels (Supplemental Fig. S4*A*), indicating that βAR stimulation did not evoke cellular ROS but specifically modulated RyR2-SNO.

Thus, overexpressed WT-S100A1 protein might not generally act as a buffer for reactive oxygen species via C85 but exert compartmentalized actions confined to S100A1 target proteins.

**Figure 7.**
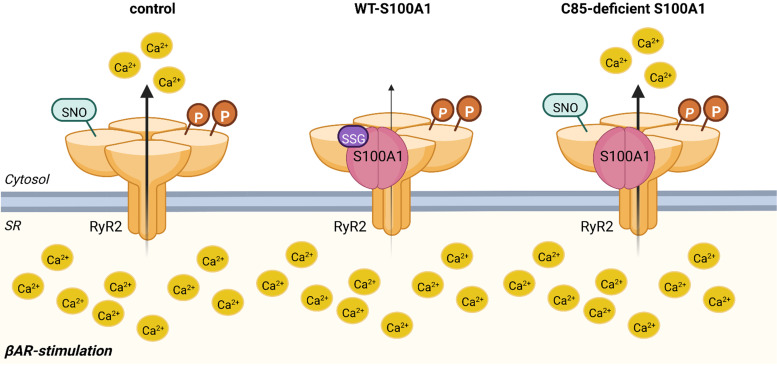
Proposed model for S100A1 regulation of diastolic ryanodine receptor 2 (RyR2) leakage. During β-adrenergic receptor (βAR) stimulation, increased phosphorylation and nitrosylation renders RyR2 leaky, resulting in diastolic Ca^2+^ release. Overexpression of S100A1 prevents RyR2 nitrosylation and, subsequently, abrogates diastolic Ca^2+^ leak. Overexpression of S100A1 variants, which lack endogenous glutathionylation sites, fails to attenuate RyR nitroslyation and have no protective effect on diastolic Ca^2+^ leakage. Created with a licensed version of BioRender.com.

Together, this let us to conclude that during diastole, under low-Ca^2+^ conditions, S100A1 interaction with the RyR2 prevents RyR2 hypernitrosylation and subsequent SR Ca^2+^ leakage during βAR stimulation, which the later one being dependent on C85 of S100A1 (Fig. 7).

## DISCUSSION

Taking advantage of engineered S100A1 protein variants, our data show for the first time that the conserved, single C-terminal C85 residue is a key moiety for S100A1’s ability to mitigate diastolic RyR2 dysfunction and, most importantly, to oppose βAR-mediated arrhythmogenic SR Ca^2+^ waves. Here, the C85 residue appears critical for protecting RyR2 against βAR-induced hypernitrosylation, which is considered to render RyR2s leaky during cardiac relaxation (30, 31, 34). As such, our study advances our understanding of the molecular mechanism underlying the previously reported antiarrhythmic efficacy of the cardiac EF-hand Ca^2+^ sensor in normal and failing CMs in vitro and in vivo (23, 25).

Our investigation originated from a series of studies that addressed the impact of C85’s ox-PTM on S100A1’s ability to transition from the inactive “apo” to the Ca^2+^-activated “holo” state (10, 12, 14). In this context, ox-PTM of C85 was demonstrated to significantly improve S100A1’s Ca^2+^ affinity by several orders of magnitude. Using an mBSA, we could confirm that S100A1 is endogenously modified in the adult heart, and specifically, glutathionylated. This is in contrast to a previous report that suggested that S100A1 is nitrosylated in neuronal cells (12). However, this observation is in line with the notion that the glutathione concentration is very high in the cell, and thus glutathionylation could be the predominant modification of S100A1 (10).

In CMs, ox-PTM of C85 might facilitate a Ca^2+^-activated state of S100A1 already at low diastolic Ca^2+^ levels and subsequently engage with intracellular target proteins (15). To test this hypothesis and determine if S100A1’s ox-PTM is required for the molecule to function properly lead us to explore the effect of S100A1 protein variants that lack C85 and ox-PTM in a series of molecular and functional assays making use of both isolated neonatal and adult rat CMs.

Analyses of SR Ca^2+^ waves and diastolic RyR2 function in CMs unveiled then the most important result of our study. Imposing significant stress on diastolic RyR2 closure, only WT-S100A1 effectively protected CMs against the high number of recurrent Ca^2+^ waves. This is congruent with recent findings demonstrating that WT-S100A1 overexpression protected both normal and failing CMs against βAR-mediated exacerbation of proarrhythmogenic diastolic Ca^2+^ waves and after contractions (23). In contrast, C85-deficient S100A1 protein variants failed to recapitulate the beneficial diastolic actions of WT-S100A1, particularly upon βAR stimulation. These results show for the first time that S100A1’s single C85 moiety seems indispensable for the protective effect of WT-S100A1 against diastolic SR Ca^2+^ leak and RyR2 dysfunction.

The hypothesis that S100A1’s single reactive cysteine residue or its NO-dependent PTM might though be required for the molecule to function properly at diastolic Ca^2+^ concentrations prompted us to assess S100A1/RyR2 interaction patterns. Our PLA study performed in resting CMs that provides diastolic-like Ca^2+^ concentrations within an intact subcellular microenvironment points toward the notion that C85-deficient S100A1 protein variants can nevertheless interact with the RyR2 to the same extent as WT-S100A1 in the relaxation phase. In agreement with these observations, it has been proposed that Ca^2+^ affinity of S100A1 might also increase upon interaction with the RyR2 and thus, that enhanced Ca^2+^ affinity might be consequence of and not prerequisite for target protein interaction (12, 35). This is also in line with the idea that S100A1 is an intrinsic disordered protein (IDP) (12). Although ordered S100A1 regions seem to dominate in the formation of the homodimeric backbone, ox-PTM of C85 is supposed to directly fine tune the hydrophobic core of S100A1, which restructures the whole protein dimer, exposing sequence fragments ascribed to target binding and subsequent activity modulation (10–12). This mechanism might unveil a flexible regulatory epitope that could also contribute to diastolic RyR2 activity modulation through a reversible S100A1/RyR2 disulfide bond formation after S100A1 binding to the RyR2 has been stabilized. However, this new hypothesis clearly warrants further validation and detailed structural studies to entail the exact binding mode of S100A1 on the RyR2.

We next focused on the phosphorylation and nitrosylation state of the macromolecular SR Ca^2+^ release channel (28, 29, 31). The Iso-induced RyR2 phosphorylation at Ser-2808 and Ser-2814 in combination with the more than doubled nitrosylation state might have significantly contributed to diastolic RyR2 dysfunction in the control group (30, 32, 34). Importantly, inhibition of βAR-mediated hypernitrosylation by S100A1 might be the critical link that conveys the protective effect of an enhanced association of the EF-hand Ca^2+^ sensor with the RyR2. These data strongly argue in favor of a critical role for S100A1’s single C85 residue in controlling the RyR2 nitrosylation state, most likely after binding has been established between both molecules.

As RyR2 hypernitrosylation is supposed to render the SR Ca^2+^ channel leaky (34, 36), we propose a model where C85’s ox-PTM of S100A1 specifically modulates diastolic RyR2 activity by controlling the channel’s redox state. This is further supported by the finding that systolic performance by overexpressed C85-deficient S100A1 protein variants was indistinguishable from WT-S100A1 protein.

Hence, this prompts the question how the single cysteine might contribute to S100A1-mediated RyR2 regulation? Aiming to corroborate a direct link between NO regulation and S100A1, we focused our effort on NOS1, which is known to regulate RyR2 activity and SR Ca^2+^ handling (32, 33). However, this link could not be established, which argues against a role for S100A1 in the control of overall CM redox homeostasis. Alternatively, spatiotemporal changes in NO and ROS have been proposed to regulate nitrosylation and subsequently activity of the RyR2 (32, 37). However, to better understand this concept, improvement of experimental methods to detect fluctuating NO levels is clearly needed.

There are some limitations associated with our conclusions. First, experiments were performed only in male CMs, or in mixed cell populations. Sex hormones affect cellular Ca^2+^ handling and the activity of ion channels (38, 39), so it remains unclear if S100A1 and C85-deficient variants have any sex-dependent effects. Second, C85-deficient S100A1 variants were overexpressed in the presence of WT S100A1. S100A1 can form heterodimers with other S100 proteins (40, 41), and we cannot rule out the formation of heterodimeric complexes between WT-S100A1 and C85-deficient mutants, but also with other S100 proteins. However, given that C85 lies outside of the binding interface (41), we do not believe that C85 variants have different affinities for homo- or heterodimer formation.

In summary, dissecting S100A1’s molecular structure-function relationship in CMs, our data indicate for the first time that the C-terminal conserved single cysteine 85 is inevitable for WT-S100A1’s antiarrhythmic but dispensable for its inotropic efficacy in CMs. Our data strongly support the notion that the protective effect of S100A1 against diastolic RyR2 dysfunction might rely on the control of the RyR2 nitrosylation state and more specifically unveil a critical role of the C85 moiety in abrogating RyR2 hypernitrosylation. Therefore, we propose a simplified two-step model where C85 seems dispensable for S100A1/RyR2 interaction but indispensable for beneficially modulating diastolic RyR2 activity. This effect could be due to the IDP properties of WT-S100A1 that requires the presence and ox-PTM of C85.

Our novel results doubtlessly advance our understanding of the molecular mechanisms underlying the beneficial pleiotropic actions of WT-S100A1 in the heart. Continued research is now needed to delineate S100A1 binding epitopes with the RyR2 and its potential involvement in the spatially confined SR nitrosylation/denitrosylation cycle. This might aid a deepened understanding of the molecular S100A1/RyR2 liaison given the therapeutic potential of S100A1 in heart failure.

## DATA AVAILABILITY

Additional data supporting the findings of this study can be found in the supplementary material (https://doi.org/10.6084/m9.figshare.25930822.v2).

## SUPPLEMENTAL DATA

10.6084/m9.figshare.25930822.v2 Supplemental Figs. S1–S4: https://doi.org/10.6084/m9.figshare.25930822.v2.

## GRANTS

This work was supported in part by grants of the German Cardiovascular Research Center DZHK 81Z0500101 (to P.M.), 81X3500143 (to J.R.), and the Klaus Tschira Foundation/Informatic for Life consortium (to P.M.).

## DISCLOSURES

P.M. and H.K. hold patents on the therapeutic use of S100A1 in cardiovascular diseases. None of the other authors has any conflicts of interest, financial or otherwise, to disclose.

## AUTHOR CONTRIBUTIONS

A.S., P.M., and J.R. conceived and designed research; A.S., J.K., A.S., S.S., A.J., and J.R. performed experiments; A.S., M.B., and J.R. analyzed data; A.S., P.M., and J.R. interpreted results of experiments; J.R. prepared figures; P.M. and J.R. drafted manuscript; A.S., H.A.K., P.M., and J.R. edited and revised manuscript; A.S., M.B., J.K., A.S., S.S., A.J., H.A.K., P.M., and J.R. approved final version of manuscript.

## References

[1] Kato K, Kimura S. S100ao (alpha alpha) protein is mainly located in the heart and striated muscles. Biochim Biophys Acta 842: 146–150, 1985. doi:10.1016/0304-4165(85)90196-5. 4052452

[2] Most P, Bernotat J, Ehlermann P, Pleger ST, Reppel M, Börries M, Niroomand F, Pieske B, Janssen PM, Eschenhagen T, Karczewski P, Smith GL, Koch WJ, Katus HA, Remppis A. S100A1: a regulator of myocardial contractility. Proc Natl Acad Sci USA 98: 13889–13894, 2001. doi:10.1073/pnas.241393598. 11717446 PMC61137

[3] Rohde D, Busch M, Volkert A, Ritterhoff J, Katus HA, Peppel K, Most P. Cardiomyocytes, endothelial cells and cardiac fibroblasts: S100A1's triple action in cardiovascular pathophysiology. Future Cardiol 11: 309–321, 2015 [Erratum in Future Cardiol 11: 502, 2015]. doi:10.2217/fca.15.18. 26021637 PMC11544369

[4] Most P, Seifert H, Gao E, Funakoshi H, Volkers M, Heierhorst J, Remppis A, Pleger ST, DeGeorge BR Jr, Eckhart AD, Feldman AM, Koch WJ. Cardiac S100A1 protein levels determine contractile performance and propensity toward heart failure after myocardial infarction. Circulation 114: 1258–1268, 2006. doi:10.1161/CIRCULATIONAHA.106.622415. 16952982

[5] Remppis A, Most P, Löffler E, Ehlermann P, Bernotat J, Pleger S, Börries M, Reppel M, Fischer J, Koch WJ, Smith G, Katus HA. The small EF-hand Ca^2+^ binding protein S100A1 increases contractility and Ca^2+^ cycling in rat cardiac myocytes. Basic Res Cardiol 97, *Suppl* 1: I56–I62, 2002. doi:10.1007/s003950200031. 12479236

[6] Ritterhoff J, Most P. Targeting S100A1 in heart failure. Gene Ther 19: 613–621, 2012. doi:10.1038/gt.2012.8. 22336719

[7] Most P, Remppis A, Pleger ST, Löffler E, Ehlermann P, Bernotat J, Kleuss C, Heierhorst J, Ruiz P, Witt H, Karczewski P, Mao L, Rockman HA, Duncan SJ, Katus HA, Koch WJ. Transgenic overexpression of the Ca^2+^-binding protein S100A1 in the heart leads to increased in vivo myocardial contractile performance. J Biol Chem 278: 33809–33817, 2003. doi:10.1074/jbc.M301788200. 12777394

[8] Boerries M, Most P, Gledhill JR, Walker JE, Katus HA, Koch WJ, Aebi U, Schoenenberger CA. Ca^2+^-dependent interaction of S100A1 with F1-ATPase leads to an increased ATP content in cardiomyocytes. Mol Cell Biol 27: 4365–4373, 2007. doi:10.1128/MCB.02045-06. 17438143 PMC1900044

[9] Yamasaki R, Berri M, Wu Y, Trombitás K, McNabb M, Kellermayer MS, Witt C, Labeit D, Labeit S, Greaser M, Granzier H. Titin-actin interaction in mouse myocardium: passive tension modulation and its regulation by calcium/S100A1. Biophys J 81: 2297–2313, 2001. doi:10.1016/S0006-3495(01)75876-6. 11566799 PMC1301700

[10] Zhukov I, Ejchart A, Bierzyński A. Structural and motional changes induced in apo-S100A1 protein by the disulfide formation between its Cys 85 residue and β-mercaptoethanol. Biochemistry 47: 640–650, 2008. doi:10.1021/bi701762v. 18088104

[11] Nowakowski M, Jaremko Ł, Jaremko M, Zhukov I, Belczyk A, Bierzyński A, Ejchart A. Solution NMR structure and dynamics of human apo-S100A1 protein. J Struct Biol 174: 391–399, 2011. doi:10.1016/j.jsb.2011.01.011. 21296671

[12] Lenarčič Živković M, Zaręba-Kozioł M, Zhukova L, Poznański J, Zhukov I, Wysłouch-Cieszyńska A. Post-translational S-nitrosylation is an endogenous factor fine tuning the properties of human S100A1 protein. J Biol Chem 287: 40457–40470, 2012. doi:10.1074/jbc.M112.418392. 22989881 PMC3504761

[13] Zhukova L, Zhukov I, Bal W, Wyslouch-Cieszynska A. Redox modifications of the C-terminal cysteine residue cause structural changes in S100A1 and S100B proteins. Biochim Biophys Acta 1742: 191–201, 2004. doi:10.1016/j.bbamcr.2004.10.002. 15590070

[14] Goch G, Vdovenko S, Kozłowska H, Bierzyñski A. Affinity of S100A1 protein for calcium increases dramatically upon glutathionylation. FEBS J 272: 2557–2565, 2005. doi:10.1111/j.1742-4658.2005.04680.x. 15885104

[15] Sun B, Kekenes-Huskey PM. Molecular basis of S100A1 activation and target regulation within physiological cytosolic Ca^2+^ levels. Front Mol Biosci 7: 77, 2020. doi:10.3389/fmolb.2020.00077. 32656226 PMC7324869

[16] He TC, Zhou S, da Costa LT, Yu J, Kinzler KW, Vogelstein B. A simplified system for generating recombinant adenoviruses. Proc Natl Acad Sci USA 95: 2509–2514, 1998. doi:10.1073/pnas.95.5.2509. 9482916 PMC19394

[17] Remppis A, Pleger ST, Most P, Lindenkamp J, Ehlermann P, Schweda C, Löffler E, Weichenhan D, Zimmermann W, Eschenhagen T, Koch WJ, Katus HA. S100A1 gene transfer: a strategy to strengthen engineered cardiac grafts. J Gene Med 6: 387–394, 2004. doi:10.1002/jgm.513. 15079813

[18] Aesif SW, Janssen-Heininger YMW, Nl R. Chapter 17—Protocols for the detection of S-glutathionylated and S-nitrosylated proteins in situ. In: Methods in Enzymology, edited by Cadenas E. Packer LAcademic Press, 2010, p. 289–296. 10.1016/S0076-6879(10)74017-9 PMC3113509 20609917

[19] Butturini E, Boriero D, Carcereri de Prati A, Mariotto S. Immunoprecipitation methods to identify S-glutathionylation in target proteins. MethodsX 6: 1992–1998, 2019. doi:10.1016/j.mex.2019.09.001. 31667096 PMC6812339

[20] Li R, Kast J. Biotin switch assays for quantitation of reversible cysteine oxidation. Methods Enzymol 585: 269–284, 2017. doi:10.1016/bs.mie.2016.10.006. 28109433

[21] Forrester MT, Foster MW, Stamler JS. Assessment and application of the biotin switch technique for examining protein S-nitrosylation under conditions of pharmacologically induced oxidative stress. J Biol Chem 282: 13977–13983, 2007. doi:10.1074/jbc.M609684200. 17376775

[22] Völkers M, Loughrey CM, Macquaide N, Remppis A, DeGeorge BR Jr, Wegner FV, Friedrich O, Fink RH, Koch WJ, Smith GL, Most P. S100A1 decreases calcium spark frequency and alters their spatial characteristics in permeabilized adult ventricular cardiomyocytes. Cell Calcium 41: 135–143, 2007. doi:10.1016/j.ceca.2006.06.001. 16919727

[23] Ritterhoff J, Völkers M, Seitz A, Spaich K, Gao E, Peppel K, Pleger ST, Zimmermann WH, Friedrich O, Fink RHA, Koch WJ, Katus HA, Most P. S100A1 DNA-based inotropic therapy protects against proarrhythmogenic ryanodine receptor 2 dysfunction. Mol Ther 23: 1320–1330, 2015. doi:10.1038/mt.2015.93. 26005840 PMC4817858

[24] Most P, Lerchenmüller C, Rengo G, Mahlmann A, Ritterhoff J, Rohde D, Goodman C, Busch CJ, Laube F, Heissenberg J, Pleger ST, Weiss N, Katus HA, Koch WJ, Peppel K. S100A1 deficiency impairs postischemic angiogenesis via compromised proangiogenic endothelial cell function and nitric oxide synthase regulation. Circ Res 112: 66–78, 2013. doi:10.1161/CIRCRESAHA.112.275156. 23048072 PMC3760372

[25] Weber C, Neacsu I, Krautz B, Schlegel P, Sauer S, Raake P, Ritterhoff J, Jungmann A, Remppis AB, Stangassinger M, Koch WJ, Katus HA, Muller OJ, Most P, Pleger ST. Therapeutic safety of high myocardial expression levels of the molecular inotrope S100A1 in a preclinical heart failure model. Gene Ther 21: 131–138, 2014. doi:10.1038/gt.2013.63. 24305416 PMC4095772

[26] Most P, Pleger ST, Völkers M, Heidt B, Boerries M, Weichenhan D, Löffler E, Janssen PM, Eckhart AD, Martini J, Williams ML, Katus HA, Remppis A, Koch WJ. Cardiac adenoviral S100A1 gene delivery rescues failing myocardium. J Clin Invest 114: 1550–1563, 2004. doi:10.1172/JCI21454. 15578088 PMC529280

[27] Eisner DA, Caldwell JL, Trafford AW, Hutchings DC. The control of diastolic calcium in the heart: basic mechanisms and functional implications. Circ Res 126: 395–412, 2020. doi:10.1161/CIRCRESAHA.119.315891. 31999537 PMC7004450

[28] Marx SO, Marks AR. Dysfunctional ryanodine receptors in the heart: new insights into complex cardiovascular diseases. J Mol Cell Cardiol 58: 225–231, 2013. doi:10.1016/j.yjmcc.2013.03.005. 23507255 PMC4042628

[29] Wehrens XH, Lehnart SE, Reiken S, Vest JA, Wronska A, Marks AR. Ryanodine receptor/calcium release channel PKA phosphorylation: a critical mediator of heart failure progression. Proc Natl Acad Sci USA 103: 511–518, 2006. doi:10.1073/pnas.0510113103. 16407108 PMC1334677

[30] Beigi F, Gonzalez DR, Minhas KM, Sun QA, Foster MW, Khan SA, Treuer AV, Dulce RA, Harrison RW, Saraiva RM, Premer C, Schulman IH, Stamler JS, Hare JM. Dynamic denitrosylation via S-nitrosoglutathione reductase regulates cardiovascular function. Proc Natl Acad Sci USA 109: 4314–4319, 2012. doi:10.1073/pnas.1113319109. 22366318 PMC3306718

[31] Nikolaienko R, Bovo E, Zima AV. Redox dependent modifications of ryanodine receptor: basic mechanisms and implications in heart diseases. Front Physiol 9: 1775, 2018. doi:10.3389/fphys.2018.01775. 30574097 PMC6291498

[32] Vielma AZ, León L, Fernández IC, González DR, Boric MP. Nitric oxide synthase 1 modulates basal and β-adrenergic-stimulated contractility by rapid and reversible redox-dependent S-nitrosylation of the heart. PLoS One 11: e0160813, 2016. doi:10.1371/journal.pone.0160813. 27529477 PMC4986959

[33] Cutler MJ, Plummer BN, Wan X, Sun QA, Hess D, Liu H, Deschenes I, Rosenbaum DS, Stamler JS, Laurita KR. Aberrant S-nitrosylation mediates calcium-triggered ventricular arrhythmia in the intact heart. Proc Natl Acad Sci USA 109: 18186–18191, 2012. doi:10.1073/pnas.1210565109. 23071315 PMC3497770

[34] Fauconnier J, Thireau J, Reiken S, Cassan C, Richard S, Matecki S, Marks AR, Lacampagne A. Leaky RyR2 trigger ventricular arrhythmias in Duchenne muscular dystrophy. Proc Natl Acad Sci USA 107: 1559–1564, 2010. doi:10.1073/pnas.0908540107. 20080623 PMC2824377

[35] Rebbeck RT, Nitu FR, Rohde D, Most P, Bers DM, Thomas DD, Cornea RL. S100A1 protein does not compete with calmodulin for ryanodine receptor binding but structurally alters the ryanodine receptor calmodulin complex. J Biol Chem 291: 15896–15907, 2016. doi:10.1074/jbc.M115.713107. 27226555 PMC4957069

[36] Xu L, Eu JP, Meissner G, Stamler JS. Activation of the cardiac calcium release channel (ryanodine receptor) by poly-S-nitrosylation. Science 279: 234–237, 1998. doi:10.1126/science.279.5348.234. 9422697

[37] Schulman IH, Hare JM. Regulation of cardiovascular cellular processes by S-nitrosylation. Biochim Biophys Acta 1820: 752–762, 2012. doi:10.1016/j.bbagen.2011.04.002. 21536106 PMC5509026

[38] Firth JM, Yang H-Y, Francis AJ, Islam N, MacLeod KT. The effect of estrogen on intracellular Ca^2+^ and Na^+^ regulation in heart failure. JACC Basic Transl Sci 5: 901–912, 2020. doi:10.1016/j.jacbts.2020.06.013. 33015413 PMC7524784

[39] Costa S, Saguner AM, Gasperetti A, Akdis D, Brunckhorst C, Duru F. The link between sex hormones and susceptibility to cardiac arrhythmias: from molecular basis to clinical implications. Front Cardiovasc Med 8: 644279, 2021. doi:10.3389/fcvm.2021.644279. 33681311 PMC7925388

[40] Rustandi RR, Baldisseri DM, Inman KG, Nizner P, Hamilton SM, Landar A, Landar A, Zimmer DB, Weber DJ. Three-dimensional solution structure of the calcium-signaling protein apo-S100A1 as determined by NMR. Biochemistry 41: 788–796, 2002. doi:10.1021/bi0118308. 11790100

[41] Wang G, Zhang S, Fernig DG, Spiller D, Martin-Fernandez M, Zhang H, Ding Y, Rao Z, Rudland PS, Barraclough R. Heterodimeric interaction and interfaces of S100A1 and S100P. Biochem J 382: 375–383, 2004. doi:10.1042/BJ20040142. 15171681 PMC1133950

